# A Lumped Parameter Modelling Study of Leukoaraiosis Suggests Its Vascular Pathophysiology May Be Similar to Normal-Pressure Hydrocephalus

**DOI:** 10.3390/brainsci15091023

**Published:** 2025-09-22

**Authors:** Grant A. Bateman, Alexander R. Bateman

**Affiliations:** 1 Department of Medical Imaging, John Hunter Hospital, Newcastle, NSW 2310, Australia; 2 College of Health, Medicine and Wellbeing, Newcastle University School of Medicine and Public Health, Callaghan Campus, Newcastle, NSW 2308, Australia; 3 College of Engineering, Science and Environment, Newcastle University School of Engineering, Callaghan Campus, Newcastle, NSW 2308, Australia; alex.bateman@newcastle.edu.au

**Keywords:** cerebral blood flow, cerebral blood volume, leukoaraiosis, normal-pressure hydrocephalus, pulsation, vascular dementia

## Abstract

Introduction: Leukoaraiosis (LA) or white matter disease is a significant component of vascular dementia. There is a large overlap noted between normal-pressure hydrocephalus (NPH) and LA. A previously reported lumped parameter modelling study of NPH led to novel findings in this disease. Given the overlap between LA and NPH, the purpose of the current study is to perform a lumped parameter study into LA to see if the vascular pathophysiology is similar to NPH. Methods: A lumped parameter model originally developed to study normal-pressure hydrocephalus was extended to investigate LA. The model was constrained by the known cerebral blood flow and cerebral blood volumes found in LA, as derived from the literature. Results: Similar to NPH, in LA, the model predicted a balanced increase in arterial and venous outflow resistance, with the resulting ischemia affecting the white matter rather than the grey matter. However, unlike NPH, in LA, the findings are irreversible, most likely due to structural venous wall changes. Conclusions: The model suggests that the vascular physiology of LA maybe similar to NPH. A common pathophysiology is discussed based on a pulsation-induced increase in the venous outflow resistance.

## 1. Introduction

Neuroimaging often shows areas of low density on CT and a high T2 signal intensity on MRI within the white matter of the brain. This change is denoted as white matter disease or leukoaraiosis (LA) [1]. The term leukoaraiosis comes from the Greek language, leuko meaning white and araiosis meaning rarefaction [2]. Pathological studies have found demyelination and reactive gliosis [3] to be the cause of the imaging findings. The most significant correlation of leukoaraiosis is with aging [4]. There is also a strong vascular component to the aetiology of LA, with risk factors of hypertension, diabetes mellitis, and cardiac disease also being prominent [5]. Indeed, leukoaraiosis is a major determinant of vascular dementia [6], which is, by definition, known to be of vascular origin. There is also an interesting overlap between normal-pressure hydrocephalus (NPH) and LA [7]. Up to 73% of patients with NPH have significant degrees of leukoaraiosis within the deep white matter [8]. This suggests a possible degree of common causation between these two disorders.

In a previous study performed by the current authors, a lumped parameter vascular model was developed to study the vascular pathophysiology of NPH [9]. This model suggested that there was a balanced increase in the vascular resistance in the arterial inflow and the venous outflow in this disease. There was a significant difference between the findings in the grey matter and white matter, with significant ischemia within the white matter in NPH [10]. Given the model’s previous findings in NPH, and the overlap between NPH and LA, the purpose of the current study is to extend the NPH lumped parameter modelling study [9], utilizing the parameters gleaned from the literature to study the vascular pathophysiology of leukoaraiosis.

## 2. Materials and Methods

### 2.1. Equations

The equations used in this study were extensively discussed previously [9] and can be reviewed in this study. However, the relevant equations will be reproduced here for convenience. Firstly, Ohm’s law for hydraulic circuits is required:

(1) ∆P=Q × R

Δ*P* is the pressure drop across the vascular segment, *Q* is the flow rate, and *R* is the resistance. Resistances in series are additive, therefore.

(2) $$R_{art}+R_{cap}+R_{ven}+R_{cuf}=R_{tot}$$

*R_art_*, *R_cap_*, *R_ven_*, *R_cuf_*, and *R_tot_* are the resistances of the artery, capillary, venous cuff, and the total system, respectively. Poiseuille’s equation calculates the pressure drop across each of these segments:

(3) ΔP=8μLQπr4

Δ*P* is the pressure drop across the vascular segment, *µ* the viscosity, *L* the vessel length, *Q* the fluid flow rate, *π* the circle proportionality constant, and *r* the radius. Given that the viscosity and vessel lengths are constants in this study, it can be shown that there is a direct relationship between a change in a segment’s resistance and a change in its volume.

(4) $$∆R=∆V^{-2}$$

where Δ*R* is the change in resistance, and Δ*V* the change in volume. It was previously shown that the venous outflow varies with the transmural pressure, as demomstrated using the following equation [9]:

(5) $${∆TMP}_{ven}=-0.033{{\Delta V}_{ven}}^{2}+7.49\times {\Delta V}_{ven}-3.44$$

Δ*TMP_ven_* is the normalized increase in venous transmural pressure, and Δ*V_ven_* is the change in venous volume.

### 2.2. Model Input Parameters

The input parameters used in this modelling are also unchanged from the previous study [9] and are summarized in Table 1. The details can be obtained from the original study. Of note, the normal sagittal sinus pressure is derived by subtracting the CSF-venous sinus pressure gradient from the ICP. The pre-outflow cuff pressure is obtained by adding the venous TMP to the ICP. The total CBV is obtained by taking the percentages of the brain made up of the grey and white matter and multiplying each by the brain weight and then the CBV for each of these components. The venous volume is obtained by subtracting the arterial and capillary volume from the total.

### 2.3. Vessel Responses to Transmural Pressure Variations

It is assumed that the changes in arterial resistance and volume in this model depend entirely on active arterial constriction or dilatation and not the vessel transmural pressure.

The capillary and venous beds are assumed to be without active constriction or dilatation [25] but vary depending on their transmural pressures. It is further assumed that a moderate reduction in capillary TMP below normal does not change the capillary size, but a maximal increase in TMP will increase their volume. The previous study indicated that an increase in capillary TMP from 12 to 37.9 mmHg would increase the capillary volume by 44%. or a 1.7% increase in volume for each 1 mmHg pressure rise. Below a TMP of 12 mmHg, the volume is unchanged at 20.3 mL, and above a TMP of 37.9, the elastic limit is reached and the volume is set to 29.2 mL [9]. The veins are assumed to alter their size purely depending on their transmural pressures. In a previous modelling study [9], the function for outflow vein dilatation was found to be summarized by Equation (5).

The outflow cuff is the segment joining the cortical veins to the sinus wall. The collapse of this segment occurs due to the transmural pressure between the ICP and the sinus pressure, which is usually negative [26]. The segment is short, and it is mostly under a state of collapse secondary to physiological ICPs. The change in volume from this segment will be ignored in this model. However, its resistance will be taken into consideration.

## 3. Results

### 3.1. Whole-Brain Findings

**Figure 1:**
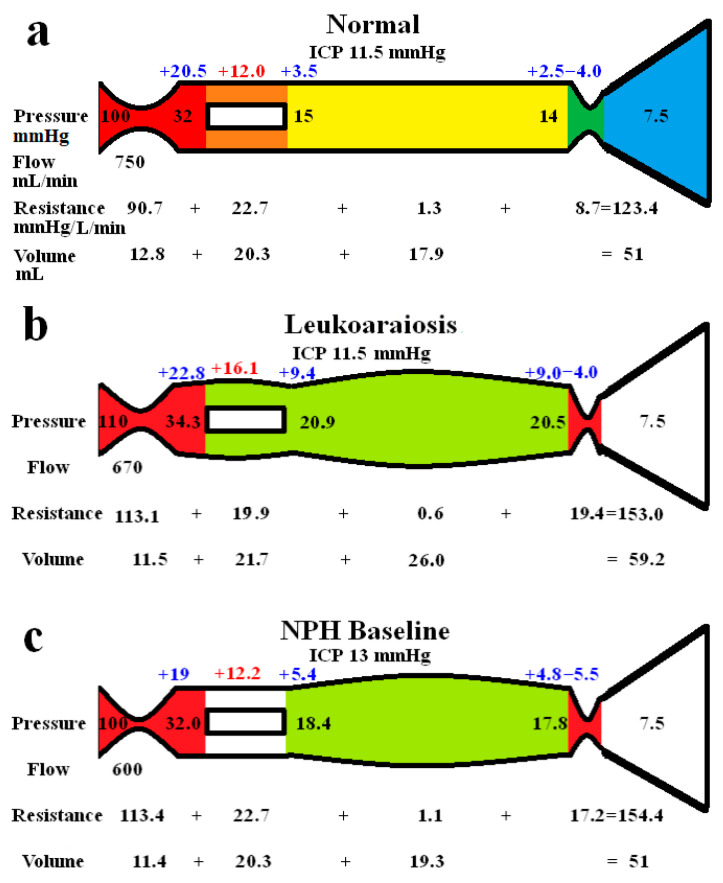
Results of modelling. (**a**) The normal findings. The segments are red for arterial, orange for the capillary, yellow for the veins, green for the outflow cuff, and blue for the venous sinus. The vascular pressures are shown within the vessels. The blue numbers are the transmural pressures at each site. The red number is the average capillary transmural pressure. The resistances and volumes for each segment are shown below the vessel. (**b**) The findings in leukoaraiosis. The red area indicates an increase in resistance in the arteries and outflow cuff, and the green decreased resistance in the capillaries and veins compared to normal. Both the capillary and venous TMP are increased, giving vascular dilatation. (**c**) The findings in NPH from a previous study. The overall findings are similar except the capillary TMP is lower and the pressure gradient across the sinus wall higher than in leukoaraiosis. Note: ICP, intracranial pressure; min, minute; and mmHg, millimetres of mercury. (**a**,**c**) are reproduced from [9] under a CC BY 4.0 commons licence.

The whole-brain modelling findings are summarized in Figure 1. The five vascular segments modelled are shown in Figure 1a, with the arterial in red, the capillaries in orange, the veins in yellow, the outflow cuff in green, and the sinus in blue. The pressures from Table 1 have been appended to each vascular segment within the vessels in Figure 1a. The resistance of each segment can be calculated using Equation (1) and is appended below the vessels in Figure 1. The normal cerebral blood volume (CBV) values are shown below the resistances. The blue numbers represent the transmural pressure gradients for each vessel segment, which are obtained by subtracting the ICP from the segment pressure. The red figure is the average capillary TMP obtained by averaging the values from before and after the capillaries. Figure 1b represents the findings in leukoaraiosis. Figure 1c was obtained from the previous modelling study into NPH [9] and is appended for comparison. In these later two figures, the red segments represent the areas of increased resistance compared to the normal findings, and the green represent reduced resistance.

In Figure 1b, the whole-brain findings in leukoaraiosis are modelled. The arterial inflow and venous outflow pressures, the ICP, the mean blood flow rate, and target CBV were obtained from the literature. The sinus pressure was assumed to be unchanged due to the normal ICP.

The pressure drop from the arteries to the sinus plus the blood flow through them allows the calculation of the total vascular resistance using Equation (1), i.e., it is increased to 153.0 mmHg/L/min compared to 123.4 mmHg/L/min in the normal model. The increase in resistance needed to be apportioned within the model. By necessity, a change in resistance changes the volume of the segment it affects. The TMP across the outflow cuff (obtained by subtracting the ICP from the sinus pressure) is normal. Therefore, the cuff outflow resistance was initially assumed to be normal. Knowing the cuff resistance and blood flow will allow for setting the blood pressure at the end of the veins by using Equation (1), which was therefore also normal. The normal TMP across the vein walls meant their volume using Equation (5) was normal. Similarly, the capillary resistance and volume were normal. In order for the total resistance to be balanced, the arterial inflow resistance needed to be set very high, but this reduced the arterial blood volume using Equation (4). The overall CBV at this stage undershot the target value of 59.2 mL by a large margin. Alternatively, placing all of the increased resistance on the venous side meant the CBV overshot the target by a large amount. Similar to the modelling we previously performed in NPH [9], a total resistance of 153.0 mmHg/L/min and a CBV of 59.2 mL constrained the model to a single solution, which was likely somewhere between an isolated increase in the arterial or venous outflow resistance. Using an iterative approach from both extremes, it was found the venous outflow resistance of 19.4 mmHg/L/min was the correct one. From this, the post-venous pressure could be calculated using Equation (1). Using this pressure, the venous CBV was calculated using Equation (4), and the resistance of this segment calculated using Equation (1). This gave the post-capillary pressure. Using the capillary tube law, the volume and resistance of this segment were calculated. The resultant volume of the capillaries was 21.7 mL. Knowing the total resistance and the other resistances, the arterial inflow resistance could be calculated using Equation (2). Figure 1b is the only valid solution to the constraints of the model, with all of the equations being satisfied. Note, there is an increase in both the arterial and the outflow cuff resistance, with a reduction in resistance of the other segments due to the increased pressures and volumes compared to normal. There is an increase in the mean capillary transmural pressure.

In Figure 1c, the findings in NPH from the previous study are appended for comparison [9]. Note, the arterial and venous outflow resistances are almost identical to Figure 1b, i.e., they are both increased over baseline, suggesting a balanced increase in the arterial and venous resistances. The overall blood flow is less in NPH, and the capillary transmural pressure is lower than in Figure 1b, being closer to the normal figure.

### 3.2. Differences Between the Grey and White Matter

**Figure 2:**
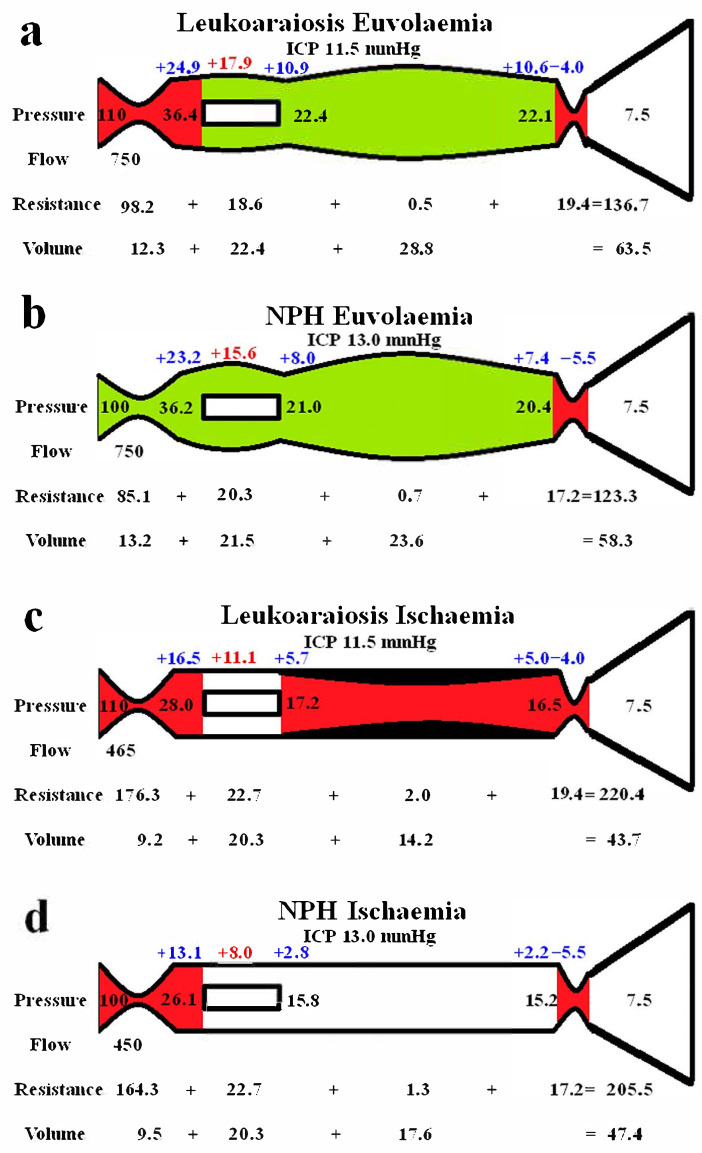
Modelling of grey and white matter. (**a**) Findings in leukoaraiosis following an increase in CBF back towards normal. There is an increase in arterial, capillary, and venous volumes, giving an overall CBV close to the target value. (**b**) Findings in NPH once the arterial inflow had been returned to normal from a previous study [10]. Note, the arterial resistance is less than in LA, but the remainder of the findings are very similar. The arterial resistance is slightly higher than normal in leukoaraiosis because of the higher inflow pressure. (**c**) Findings in leukoaraiosis once the CBF was reduced by 38%. The target CBV could not be attained unless the venous wall was thickened, giving an overall 50% reduction in venous volume compared to (**a**). (**d**) Findings in NPH from a previous study where the CBF was reduced. The overall findings are similar to (**c**), except the requirement for venous wall thickening as seen in (**c**) is not required in (**d**). (**b**,**d**) reproduced from [10] under a CC BY 4.0 commons licence.

Figure 2 shows extended modelling to gauge the differences between the grey and white matter. The red segments represent the areas of increased resistance compared to the normal findings, and the green represent reduced resistance. In Figure 2a, the brain is modelled as if all of it was being affected in a way similar to the grey matter in LA. In the study by Markus et al. [27], the grey matter CBF was normal, and the CBV was increased by 28% in LA. We elected to leave the outflow resistance as unchanged from the previous global model (Figure 1b) and reduced the arterial resistance until the CBF increased back to normal at 750 mL/min. The result was a further dilatation of the capillary and venous beds, with an increase in the capillary and venous transmural pressures. The CBV was increased to 63.5 mL, which was only 2.8% less than the target of value of 65.3 mL, as suggested by Markus et al. [27]. Figure 2b indicates the findings when the blood flow was increased to normal in NPH, similarly to Figure 2a. The findings are very similar, except there is a slight increase in arterial resistance in LA. This is required to balance the increased inflow pressure. Figure 2c shows the findings for the white matter, with increased resistance throughout the vascular system, excluding the capillaries. Some venous wall thickening and stenosis was required, or the low CBV could not be attained. The wall thickening reduced the venous CBV to a level where the total CBV could be reached. Figure 2d is the findings in the white matter in NPH, which are similar overall. Note, no venous wall thickening was required to match the target CBV.

## 4. Discussion

In this study, we have applied a lumped parameter model, previously developed to investigate NPH, to leukoaraiosis. The overall findings suggest that the vascular pathophysiology of LA may be similar to NPH. We have made many assumptions in this lumped parameter modelling study. We have tested the modelling we have performed by comparing the outcomes predicted by the model with the available literature. Previously, the model successfully predicted the cerebral blood volume changes found in the literature at the limits of autoregulation, in both human and animal studies [9]. The model also correctly predicted the cerebral blood volume changes in Alzheimer’s disease [28] and in idiopathic intracranial hypertension [29].

### 4.1. Global Brain Changes in Leukoaraiosis

The global findings within the brain are summarised in Figure 1b. In a study by Markus et al., when using an MRI contrast first-pass technique, the cerebral blood flow (CBF) in the grey matter was normal, but in the white matter, the CBF was reduced by 38% [27]. Similarly, another study showed a normal CBF in the grey matter of leukoaraiosis patients without dementia [30]. Given that the grey matter averages 65% of the brain volume and the white matter 35% [22], we can calculate the global reduction in CBF in LA to be just over 10%. This figure is comparable to a study by Henry-Feugeas et al., where the global reduction in CBF in leukoaraiosis was found to be 6% by using an MRI phase-contrast flow quantification technique [31]. Thus, in the model, a CBF of 670 mL/min was used for the blood flow rate. The mean arterial pressure in patients with significant leukoaraiosis is 10% higher compared to those with minimal disease [32], so we have increased the arterial inflow pressure to 110 mmHg in the model. The ICP in NPH patients without LA (those patients had a normal ICP) was not significantly different compared to those with mild, moderate, or severe LA [33], so we have left the ICP unchanged in the model. Given that the ICP is normal in isolated LA, the venous sinuses are unlikely to be compressed, so the venous sinus pressure was left unchanged from normal. The cerebral blood volumes (CBVs) in LA found in the study by Markus et al. showed an increase of 28% in the grey matter but a reduction of 14% in the white matter [27]. The latter result failed to reach significance, but this was most likely due to this part of the study being under-powered and unable to confirm this finding. However, a study using positron emission tomography CT showed similar results to those of Markus et al., with a reduced white matter CBV of 15% [34]. Given that the CBV of the normal grey matter is 4.6 mL/100 g, and in the white matter, it is 1.3 mL/100 g [21], and given the percentage of the brain volume that each of these two components makes up (as already discussed), the global CBV would be increased by 16.1% in LA, so we used this as our target value. The CBF and CBF values constrained the model, meaning the findings in Figure 1b were the only ones that allowed all of the equations to be satisfied. Note, there is an increase in perfusion pressure due to the increased arterial inflow pressure. The model predicts that the arterial resistance in LA is increased by 24.7% above normal. Normally, an increase in the perfusion pressure from an increase in arterial pressure constricts the arterioles (as found in the model) and therefore also reduces the global CBV [35]. However, the global CBV is elevated in LA. Therefore, we required an increase in the venous outflow resistance to dilate the veins and capillaries in order to resolve the equations successfully. This outflow resistance increase appears to correlate with the literature. An increased diameter of the basal veins of Rosenthal and internal cerebral veins is associated with the white matter hyperintensity volume in LA [36]. In another study, the internal cerebral veins were enlarged, which was related to both aging and increased white matter hyperintensity independently [37]. Using an MRI susceptibility sequence, the number of pixels within the deep white matter (assumed to be within the larger deep medullary veins) was increased by 40% in leukoaraiosis, compared to controls and correlated with white matter disease volumes [32]. Given that the dilatation within the veins of the brain also extends into the subarachnoid space component, the venous outflow stenosis producing the dilatation must be between the distal ends of the veins and the sinuses.

It can be seen from Figure 1 that there are similarities between LA and NPH. There is an overall reduction in CBF compared to normal, which is slightly more pronounced in NPH than in LA. There is a 25% increase in arterial resistance compared to normal in both diseases and a 123% increase in venous outflow resistance in LA, which is more pronounced than the 98% increase in venous outflow resistance in NPH. The differences between the diseases are that NPH appears to be associated with a CSF outflow resistance increase, manifest as an increased pressure gradient across the arachnoid granulations within the sinus wall as compared to no change in LA. The capillary transmural pressure is higher in LA than NPH, which may bring about an increase in the overall CSF formation rate compared to NPH if the blood–brain barrier were to be disrupted in the former [9]. Patients with leukoaraiosis showed a 34% increase in the CSF-to-serum albumin ratio, signifying a breakdown in the blood–brain barrier [38]. This finding was independent of the degree of dementia, atrophy, or presence of cerebral infarction. Similarly, an MRI contrast technique indicated there was significant BBB breakdown in LA [39]. The lack of a significant increase in ICP in LA despite the BBB’s expected increase in interstitial fluid leakage would suggest that the CSF outflow absorption is not deficient, unlike in NPH.

### 4.2. Differences Between the Cortex and White Matter

Figure 2 highlights the expected differences in physiology between the cortical grey matter and the deeper white matter. Figure 2a models the effect of increasing the blood flow back to normal, as per the findings within the LA literature in the grey matter, as already discussed. The outflow cuff resistance was unaltered from the previous model, and the effect was to dilate the arteries, capillaries, and veins, with an increase in total CBV from 59.2 mL in the original LA model to 63.5 mL. Thus, the euvolaemia model predicts the CBV to be only 2.8% less than the target value of 65.3 mL, as suggested by Markus et al. [27]. This tends to indicate that the modelling may be accurate enough for the current purposes. Figure 2b gives the results from previous modelling where the CBF was returned to normal in NPH [10]. The overall findings are very similar to Figure 2a, with the only significant difference being the 8.3% increase in arterial resistance in LA compared to normal. This mostly comes about due to the increased perfusion pressure from the increased arterial inflow pressure. It can be seen that LA is likely not predominately a disease of the cortex, despite the increased venous and capillary pressures, but is probably a disease of the white matter. Illustrating this, Brown et al., using Xenon CT before and after acetazolamide vasodilatation, found that the cortical blood flow increased normally in leukoaraiosis patients, but there was no significant increase in the white matter blood flow, indicating an exhaustion of autoregulation in the deeper regions and its preservation superficially [40]. However, the 61% increase in the cortical venous volume in LA as compared to normal may be expected to narrow the venous perivascular spaces [41] and thus impede the glymphatic outflow, as occurs in NPH [42]. Glymphatic disruption is noted to be a feature of LA [43], like NPH. The glymphatic flow is thought to pass from the arterial perivascular spaces, through the interstitium, and out via the venous perivascular spaces [44]. Most of the evidence suggests that the dilated perivascular spaces are around arteries and not the veins [44]. This would indicate that either there is a significant increase in glymphatic fluid inflow into the arterial perivascular spaces, which the outflow cannot handle (unlikely as the total glymphatic flow is not increased), or that the outflow passageway is obstructed, backing up the fluid further upstream. Dilatation of the veins within their perivascular spaces could provide this downstream obstruction.

Figure 2c illustrates the effect of reducing the CBF to match the reduction in blood flow found in the white matter in leukoaraiosis. The modelling was unable to reach the target CBV unless there was also wall thickening and stenosis added to the venous segment. Moody et al. studied the deep medullary veins of the brain from 20 to 800 µm diameter and found significant wall thickening in LA [45]. This thickening was termed venous collagenosis and was due to a large amount of collagen deposited within the walls. They found that 65% of subjects older than 60 years had at least a 50% stenosis of their periventricular veins [45]. They suggested that severe stenosis or occlusion of the deep cerebral veins may promote the development of leukoaraiosis [45]. In another study, collagenosis of small and medium-calibre veins was significantly worse in patients with larger volumes of LA [46]. In fact, the strongest predictor of LA score was stenosis of the large-calibre veins [46]. Venous collagenosis is a major difference between LA and pure NPH, with the NPH ischaemia model in Figure 2d not requiring wall thickening or stenosis. In those with mixed disease (LA and NPH), the LA is associated with more severe symptoms but may not affect the CSF shunting outcome [33].

In both NPH and LA, there was a large increase in arterial inflow resistance required in the white matter, despite the venous resistance increase. In LA, of the 97 mmHg/L/min increase in the white matter vascular resistance, 88.2% came from the arterioles, 11% from the venous outflow cuff, and only 0.7% from the venous bed, with the capillaries being unchanged in resistance. A major difference between NPH and LA is the reversibility of the reduced blood flow and presumably the reversibility of the large increase in arterial resistance. In NPH, there was a 53% increase in CBF in those who improved with shunting, indicating retained autoregulation [47]. This suggested to us that the brain was electing to limit the CBF in NPH, perhaps to minimize the ICP by reducing the CSF formation rate increase inherent when there is an opening of the blood–brain barrier [9,10]. There is no apparent residual autoregulation within the white matter in LA [40]. This would suggest that there may be some irreversible arterial disease as well as venous collagenosis in the deep white matter in LA. Some authors suggest that arteriolosclerosis is almost always detected within areas of LA [48], but not all studies show such a strong correlation. Both arteriolosclerosis and venular collagenosis are more prevalent with age. However, arterial sclerosis was associated with lacunar infarction and haemosiderin deposition but not leukoaraiosis severity in one study [49]. Conversely, venular collagenosis was not associated with lacunes or haemosiderin but was associated with leukoaraiosis in the same study [49]. In another study, arterial wall collagen deposition was not a predictor of white matter disease, but venous collagen deposition was such a predictor [50]. Brown et al. indicated that the increase in resistance may be due to arteriolar tortuosity rather than sclerosis of the wall [51]. The results of a meta-analysis suggest that although there is lower blood flow in LA, the findings did not strongly support causation. The results suggested that the reduced flow may reflect a reduction in supply required by the tissue secondary to decreased neuronal activity or atrophy [52]. The findings indicate that there may be a functional arterial cause rather than a structural cause for the lack of irreversibility of the reduced white matter blood flow in LA.

### 4.3. Pulsatility as a Cause of Leukoaraiosis

It is apparent that there is a reduction in blood flow to the white matter in LA, but as discussed, this may not be the causative factor. Previously, one of the current authors developed a theory suggesting that there was a common pathophysiology between Alzheimer’s disease, vascular dementia, and NPH based on intracranial pulsation energy dissipation [53]. It was suggested that there was a spectrum of pulsation differences between these diseases, with the process termed pulse wave encephalopathy. The literature appears to support this hypothesis, as the percentage of the brains volume affected by LA increases in accordance with arterial pulse pressure quartiles in male subjects [2]. The severity of LA correlates with the middle cerebral artery pulsatility and the pulse wave velocity (a measure of aortic arterial stiffness) in multivariate analysis [54]. The authors concluded that increased arterial stiffening causes increased transmission of enhanced aortic pulsatility to the cerebral circulation, causing LA due to either decreased perfusion in diastole, increased endothelial shear stress, or impaired autoregulation [54]. However, the increased pulsation is not just limited to the arterial tree. Patients with dementia and moderate leukoaraiosis showed a 69%, 48%, and 34% increase in pulsatility in the blood flow of the arteries, sagittal sinus, and superficial cortical veins compared to dementia patients without leukoaraiosis [55]. In vascular dementia patients, the absolute pulsation volume of the straight sinus (which drains the deep white matter) is increased by 57% compared to normal elderly controls [56]. These venous findings correlate with NPH, where there was a 56% increase in the pulsatility index of the arteries and a 70% increase in this metric within the sagittal sinus compared to dementia patients without leukoaraiosis [55]. In NPH, the CSF pulse pressure increases by 6–8 times what is normal [57]. The CSF pulse pressure has not been measured in LA, but given the arterial, cortical vein, and sinus pulsation findings, it is likely to be increased similar to NPH. Interestingly, when veins are used as arterial bypass grafts and experience pulsatile flow, they become thickened similar to the collagenosis already discussed. In a porcine venous graft model, after the first week, wall thickening occurs largely due to extracellular matrix deposition (fibrosis) and neointimal smooth muscle cell proliferation [58]. Could pulsation energy deposited within the walls of the veins be behind the venous collagenosis noted in white matter disease as well?

The modelling highlights an apparent paradox. The previous NPH modelling suggested that a 37% increase in the pressure gradient across the outflow cuff led to a 98% increase in outflow resistance of this segment (see Figure 1c) [9]. In the current modelling, a normal pressure gradient across the outflow cuff in LA still resulted in a 123% increase in resistance of this segment (see Figure 1b). Where does this increase in resistance come from? We have previously suggested that this may be due to the effect of the CSF pulsation pressure inducing a phenomenon in the veins known as impedance pumping [59]. However, there may be a simpler explanation for this finding. The cortical veins are a series of collapsible tubes in which the pressure external to the tubes (ICP) exceeds the sinus pressure. This arrangement represents a Starling resistor. The collapse of the distal end of a Starling resistor maintains the pressure across the tube wall upstream to the collapse. This upstream transmural pressure (TMP) it is only very slightly positive in classical Starling resistors [60]. Thus, if the ICP were to increase, the venous TMP would be expected to remain nearly constant and the venous pressure to be minimally above the ICP [60]. It is apparent from the modelling that cortical veins do not act as traditional Starling resistors in this regard. The normal vein TMP is 2.5 mmHg (not close to zero as an ideal Starling resistor model would predict) and increases to 4.8 mmHg in our NPH model (see Figure 1c). It further increases to 9 mmHg in LA in our model. If the veins were ideal Starling resistors, then the upstream venous pressure would normally be just above the ICP, i.e., 11.5 mmHg. This would give a normal venous cuff resistance of 5.3 mmHg/L/min, but we estimate this resistance to be 8.7 mmHg/L/min in the normal model. The CSF pulse originates from the intracranial arterial pulse. It is dissipated by shifting CSF backwards and forwards through the foramen magnum and by compressing the cortical veins, making the venous outflow in the sagittal sinus pulsatile [55]. This sinus pulsation occurs due to compression of the outflow cuff. In MRV studies of humans with elevated ICP, 80% showed a narrow segment of the cortical vein, approximately 5 mm long at the level of the cuff. This appeared in only 10% of the control subjects [61]. The diameter of the cortical veins increased by 30% upstream from the cuff in those patients with raised ICP compared to controls, suggesting an increase in venous outflow resistance over and above that required to keep the veins open [61]. Increasing the ICP increases the CSF pulse pressure due to the intracranial compliance being reduced [62], suggesting that there may be a correlation between the ICP pulse pressure and venous outflow resistance. The peak flow in the cortical veins lags behind the peak flow in the sinus by 100 mS [55] despite the sinus being distal to the veins that drain into it. This suggests that cuff pulsation delays cortical vein emptying by generating a pressure wave going back towards the capillaries [55]. It may be envisaged that a rhythmic contraction of the vein cuff, which reduces its volume by 50% below its mean value and then increases its volume by 50% above the mean, would increase and decrease the resistance of the outflow by the same amount, and therefore they would cancel each other out. However, this may not be the case. The volume of a cylinder changes as the square of the radius, but Poiseuille’s Equation (3) indicates that the outflow resistance varies with the inverse fourth power of the radius. Therefore, a systolic reduction in venous cuff volume of 50% would increase its outflow resistance by 4 times, but an increase in volume by 50% in diastole would decrease its resistance by only 0.44 times. Given that systole lasts for 40% of the cardiac cycle and diastole for 60% on average [63], the time-averaged cuff resistance over the entire cardiac cycle would be increased by 1.9 times normal with such a pulsation. Reducing the average cuff volume by 2/3 in systole and increasing it by 2/3 in diastole with a larger pulse pressure would increase the average outflow resistance by 4.9 times, suggesting that increasing the pulsation pressure may increase the venous outflow resistance. Thus, we suggest that an increase in the CSF pulse pressure in both NPH and LA would increase the outflow resistance and pressure and send a pressure wave back toward the capillaries. In LA, we suggest that this pulsation energy induces vein wall thickening, making the walls less compliant and propagating the pressure waves further towards the capillaries with a greater amplitude [55].

### 4.4. Clinical Utility

If we are correct and the CSF pulsations generate both an increase in the venous outflow pressure and a pulse pressure wave travelling back towards the capillaries, then this could explain the blood–brain barrier disruption in both diseases. A mouse model indicates that an increase in venous pressure will disrupt the blood–brain barrier without any other requirement [64]. Therefore, in order to limit the effects of this pulsation energy and the progression of LA, strategies aimed at reducing the pulsation amplitude in both the arterial inflow and the subarachnoid space could have therapeutic value. It has been suggested that syringomyelia of the cord (the development of a cystic cavity) secondary to a Chiari I malformation (foramen magnum being blocked by the cerebellum) is analogous to NPH [65]. There is a reduction in the spinal canal subarachnoid space compliance, increased CSF pulse pressures, and disruption of the blood spinal cord barrier in syringomyelia similar to NPH [65]. A dog model of syringomyelia showed dilatation of the venous outflow of the cord, similar to our findings in both NPH and LA [66]. A posterior fossa decompression increases the compliance of the spinal canal, reduces the CSF pulse pressure, and collapses the syrinx [65]. Interestingly, increasing the cranial compliance by posterior fossa decompression also improves Chiari I-associated hydrocephalus in 90% of cases without further treatment [67]. Similarly, subtemporal craniectomy is noted to significantly reduce the ventricle size in patients with shunt-dependant hydrocephalus [68]. Increasing the intracranial compliance by craniectomy would reduce the ICP pulse pressure. Could the same procedure halt the progression of LA?

### 4.5. Limitations

Poiseuille’s equation requires that a Newtonian fluid should pass through a thin, rigid, circular tube, without turbulence, to be valid. If these assumptions hold, the findings would be accurate. However, despite the equation’s limitations, it is commonly used in modelling the vasculature within the medical literature.

We have assumed the mean arterial inflow pressure to be increased in LA to 110 mmHg. We performed a sensitivity analysis to gauge any error induced by this assumption, by increasing the arterial inflow pressure to less than the cut-off for autoregulation failure at 150 mmHg [25]. We found the capillary pressure to be unchanged, because the arterial resistance must increase to keep the blood flow from increasing [29].

We assumed the ICP in leukoaraiosis to be unchanged from normal at 11.5 mmHg, but this was derived from a single reference [33]. We performed a sensitivity analysis by increasing the ICP by 9% to 12.5 mmHg to gauge the effect this would have on the model. The result was that the venous outflow cuff resistance needed to increase by 8% and the arterial resistance reduce by 0.9% for the target CBV increase of 16.1% to be reached. This does not materially change the findings of the model, which indicate that venous outflow resistance must be increased in leukoaraiosis.

We assumed that the sinus pressure was normal at 7.5 mmHg in our model. Given that the blood flow through the sinuses was 10% lower than normal, one could argue that the sinus pressure could be less than normal if the resistance to blood flow through the venous sinuses and neck was unchanged. A sensitivity analysis was performed by reducing the sinus pressure by 13% to 6.5 mmHg. This increased the required total resistance in the system by 1% to 154.5 mmHg/L/min. In order for the target CBV increase of 16.1% to be reached, the outflow cuff resistance needed to be increased by 15% and the arterial resistance reduced by 0.9%. Again, this does not materially change the finding that the outflow cuff resistance must be increased in leukoaraiosis for the CBV to be increased.

The model that assumes the capillary volume is unchanged as the capillary TMP is reduced below normal. This assumption was made because the critical buckling pressure for such a small tube as a capillary is very high and is never approached in this model. The critical transmural pressure at which buckling of a collapsible tube begins depends on several variables, such as the stiffness of the wall (Young’s elastic modulus), Poisson’s ratio (how the wall deforms under a tensile load), the wall thickness, the internal diameter of the vessel, and the vessel length [69]. The most important of these is the ratio of the wall thickness to the internal diameter, because the critical buckling pressure is proportional to the cube of this ratio. The buckling pressure is linearly related to the other variables [69]. The average internal diameter of a human cerebral capillary is 8 μm, and the wall thickness is 1 μm [70], giving a wall thickness-to-internal diameter ratio of 0.13. The average human cortical vein adjacent to the sagittal sinus has an internal diameter of 3.3 mm and a wall thickness of 0.044 mm [59], giving a ratio of 0.013 (tenfold less). The cube of the ratio of thickness divided by diameter for the capillaries compared to the veins is therefore 1000. The elastic modulus for the capillaries is 0.68 MPa [70], and for cortical veins, it is 0.16 MPa [59], indicating that the capillary walls are stiffer than the veins. Poisson’s ratio for the tensile deformation in most materials varies between 0 and 0.5, with the value for blood vessels being 0.4 [70]. The capillaries are shorter than the cortical veins, making them harder to collapse from this viewpoint. Given that the critical buckling pressure is proportional to the cube of the thickness-to-diameter ratio, and the capillaries are stiffer and shorter than veins, then the capillaries and smallest veins will have a critical buckling pressure that is at least 1000 times greater than the distal cortical veins. The buckling pressure is probably very low in cortical veins, standing at approximately 0.1 mmHg in a dog model using the inferior vena cava [71]. The figure for capillaries will therefore be much larger, approximating 100 mmHg.

Some of the data we required is not available from human studies, and so animal studies were utilised. The data linking dilatation of the capillaries to the changes in TMP was taken from rodent studies, and the normal venous TMP was obtained from a primate study. We have no way of knowing if the animal data closely approximates human findings, so this is a limitation.

The modelling does not attempt to stratify the changes in the parameters that may occur due to differing burdens of white matter disease. This is because there is no published data to give the required CBV for the differing levels of LA. Interestingly, the reduction in CBF does not appear to be directly related to the degree to LA, with one study finding that there was no relationship between LA severity and measures of arterial inflow [72] and another indicating no clear change in CBF with the Fazekas score [55].

## 5. Conclusions

Our modelling suggests that the vascular physiology of LA may be similar to NPH. The model required thickening of the venous walls in LA, which was not required in NPH. The increase in venous outflow resistance can be explained by pulsation-derived compression and dilatation of the outflow cuff.

## Figures and Tables

**Table 1:** Summary of the normal input parameters.

Parameter	Value	Reference
Brain size	1500 g	Bell et al. [11]
Cerebral blood flow	50 mL/100 g/min	Lassen et al. [12]
Arterial inflow volume	750 mL/min	Buijs et al. [13]
Mean arterial pressure	100 mmHg	Ursino [14]
Pre-capillary bed pressure	32 mmHg	Salmon et al. [15]
End-capillary bed pressure	15 mmHg	Cirovic et al. [16]
CSF pressure	11.5 mmHg	Fleishmann et al. [17]
Pressure gradient CSF-SSS	4 mmHg	Benabid et al. [18] Pollay et al. [19]
Sagittal sinus pressure	7.5 mmHg	Bateman et al. [9]
Cortical vein TMP	2.5 mmHg	Johnston et al. [20]
Pre-outflow cuff pressure	14 mmHg	Bateman et al. [9]
Grey matter CBV	4.6 mL/100 g	Helenius et al. [21]
White matter CBV	1.3 mL/100 g	Helenius et al. [21]
Brain grey matter percentage	65%	Good et al. [22]
Brain white matter percentage	35%	Good et al. [22]
Total CBV	51 mL	Bateman et al. [9]
Arterial volume	12.8 mL	Hua et al. [23]
Capillary volume	20.3 mL	Menéndez González [24]
Venous volume	17.9 mL	Bateman et al. [9]

Note: CBV, cerebral blood volume; SSS, superior sagittal sinus; TMP, transmural pressure.

## Data Availability

The original contributions presented in this study are included in the article. Further inquiries can be directed to the corresponding author.
